# The Applicability of the Results in the Asian Population of ORIENT-11 to a Western Population According to the ICH-E5 Framework

**DOI:** 10.3389/fonc.2022.859892

**Published:** 2022-06-10

**Authors:** Stephen V. Liu, Misako Nagasaka, Victoria Stefaniak, Kristi Gruver, Yong Lin, David Ferry, Mark A. Socinski, Li Zhang

**Affiliations:** ^1^Lombardi Comprehensive Cancer Center, Georgetown University, Washington, DC, United States; ^2^Chao Family Comprehensive Cancer Center, University of California Irvine School of Medicine, Irvine, CA, United States; ^3^Oncology, Eli Lilly and Company, Indianapolis, IN, United States; ^4^Hematology and Oncology, AdventHealth Cancer Institute, Orlando, FL, United States; ^5^Medical Oncology Department, Sun Yat-sen University Cancer Center, State Key Laboratory of Oncology in South China, Collaborative Innovation Center for Cancer Medicine, Guangzhou, China

**Keywords:** sintilimab, non-small cell lung cancer, chemo-immunotherapy, immunotherapy, Asian, ORIENT-11

## Abstract

Sintilimab combined with pemetrexed and platinum met the primary endpoint of improving progression-free survival (PFS) as a first-line therapy for nonsquamous non-small cell lung cancer (NSCLC) in the phase 3 trial ORIENT-11 (NCT03607539). As seen in similar trials, the addition of sintilimab, a PD-1 inhibitor, to chemotherapy improved the PFS without significantly worsening the toxicity, with improvements in response rate and duration of response. In contrast to previous trials, the ORIENT-11 trial was conducted completely in China. Both intrinsic and extrinsic factors are important to consider when reviewing foreign clinical trial data, as they may influence the efficacy and the safety outcomes. Here we discuss the applicability of ORIENT-11 clinical results to a Western population.

## Introduction

Anti-PD (L)1 inhibitors, either alone or with chemotherapy, have emerged as the superior and preferred first-line treatment option for advanced or metastatic nonsquamous non-small cell lung cancer (NSCLC) without actionable genomic tumor alterations (1–3). Multiple phase 3 studies have shown that the addition of checkpoint inhibitors, such as pembrolizumab, atezolizumab, and nivolumab + ipilimumab, to standard chemotherapy improves both progression-free survival (PFS) and overall survival (OS) compared to chemotherapy alone for advanced NSCLC (3–6). Sintilimab is a recombinant fully human immunoglobulin G (IgG4) anti-PD-1 monoclonal antibody which has demonstrated both preclinical activity as monotherapy (7, 8) and clinical benefit when used in combination with pemetrexed and platinum chemotherapy for NSCLC (9). The ORIENT-11 trial demonstrated improved OS, PFS, and objective response rate (ORR) with the addition of sintilimab to pemetrexed plus platinum (10). The ORIENT-11 results support sintilimab plus platinum and pemetrexed as a novel first-line treatment option for nonsquamous NSCLC. This trial was conducted entirely in a Chinese population. How does this (and should this) influence the applicability of the clinical data?

Difference in trial populations should not be ignored. In the right context, they impact the applicability of trial data to real-world practice. However, clinical trial data should not be disregarded based solely on the geography of study conduct; in its extreme, this perspective reflects a form of implicit bias. It is more appropriate to identify the relevant differences between trial populations and understand their influence on outcomes. Bridging studies can provide a link between existing treatment strategies and new geographical regions. In 1998, the International Conference on Harmonization of Technical Requirements for Registration of Pharmaceuticals for Human Use (ICH) issued an E5 document entitled “Ethnic Factors in the Acceptability of Foreign Clinical Data” to help provide the framework for isolating intrinsic and extrinsic factors on efficacy and safety. Intrinsic factors include genetic, pharmacogenomic, and physiologic differences; extrinsic factors are more cultural, environmental, or societal differences. Understanding the influence of these factors will help in removing the redundant repetition of clinical trials (11).This ICH-E5 guideline supported a three-step process for determining the acceptability of foreign clinical data, which included completeness of clinical data package, sensitivity to ethnic factors, and likelihood that extrinsic factors influence a medicine’s efficacy and safety. Using the framework set forth in the ICH-E5 document, we assessed whether the foreign data from ORIENT-11 trial were applicable to the US population and US medical practice.

Herein, we will assess the key intrinsic (e.g., genetic, pharmacogenomic, and physiologic differences) and extrinsic (e.g., treatment guidance, second-line treatment, and staging and evaluation comparable between China and US) factors which support the extrapolation of the ORIENT-11 results in a Chinese population to a Western population in the treatment of first-line nonsquamous NSCLC with sintilimab.

## Results

### Mechanism of Action and Class Effect of Anti-PD-1/PD-L1 Inhibitors

Sintilimab reacts with PD-1 in line with the other PD-1 or PD-L1 agents, confirming a similar mechanism of action (4, 5, 9, 12–14). The anti-PD-1 agents pembrolizumab, sintilimab, cemiplimab, and nivolumab all share IgG4 backbones which are associated with a very low effector function and weak affinity to Fc receptors for IgG, thereby reducing the likelihood of complement-dependent cytotoxic responses and making them ideal for use in therapeutic antibodies (15–17). The monoclonal antibodies inhibit the PD-1/PD-L1 pathway, which results in antibody-dependent cell cytotoxicity and T lymphocyte stimulation. Atezolizumab does this by targeting the PD-1 ligand, whereas nivolumab, pembrolizumab, and sintilimab target the PD-1 protein and subsequently inhibit its binding with the PD-1 ligand(s) (18).

Sintilimab has demonstrated high-affinity and high-specificity binding to PD-1 *in vitro*, with blocking potency against both PD-L1 and PD-L2 and a dissociation constant of 74 pM *versus* 3,186 and 1,785 pM for nivolumab and pembrolizumab, which was confirmed independently (7, 8, 19). The antibody acts quickly, occupying the PD-1 receptors within 24 h of administration. The PD-1 receptor occupancy was high (mean ≥95%) with slow dissociation kinetics (>90% maintained over 3 weeks) (7, 20). Furthermore, in a mixed lymphocyte reaction assay, sintilimab increased interleukin-2 and interferon-γ in a dose-dependent manner, similar to nivolumab; however, sintilimab did not induce cytokine release (8). Gene expression analyses evaluating the association between clinical outcome with sintilimab and a specific immune cell signature revealed that a longer PFS and/or OS was attained in patients who had a higher MHC-II-associated gene expression (10), which has also been previously observed with pembrolizumab and nivolumab (21, 22). This potentially implicates antigen presentation pathways, such as MHC-II pathway, in the mechanism of action and a crucial component to attain clinical benefit from sintilimab combination therapy (10).

A higher incidence of *EGFR* mutations has previously been observed in Asian *versus* non-Asian populations (23). However, across anti-PD-1 studies in patients with NSCLC who do not have a driver mutation, the outcomes are comparable between Asian and Western patients (4, 9). The CheckMate 078 study assessed the efficacy of nivolumab following progression on platinum-doublet chemotherapy in a predominantly Chinese population (24). At 2 years, the results revealed long-term survival outcomes comparable with the global populations analyzed in CheckMate 017 and 057 studies (24, 25). The global KEYNOTE-042 study with 30% of its participants from East Asia also provides evidence of the comparable survival benefit afforded by pembrolizumab across both Asian and non-Asian patients (1). The KEYNOTE-189 Japanese extension study also demonstrated that the results in the Japanese population were consistent with the results in the overall population of patients (26).

An FDA meta-analysis of 11 randomized NSCLC trials provided additional evidence of this class effect observed with anti-PD-1/L1 inhibitors, demonstrating a similar treatment benefit between Asian and non-Asian populations (27). These 11 studies were divided into three groups based on the line of therapy: first-line monotherapy, first-line combination with chemotherapy, and second-line monotherapy. Overall, efficacy was similar between Asian and non-Asian patients regardless of prior lines of therapy. The OS hazard ratios (HRs) were 0.72 (0.48, 1.07) and 0.68 (0.60, 0.78), and the PFS HRs were 0.72 (0.55, 0.96) and 0.62 (0.56, 0.69) for the first-line treatment of NSCLC in Asian and non-Asian groups, respectively (27). Although the number of Asian patients in the pooled analysis is limited, the relatively consistent efficacy across both first-line and second-line NSCLC studies suggest a similar efficacy between Asian and non-Asian patients (27).

### Data From Sintilimab Studies

The double-blind, randomized, phase 3 ORIENT-11 study compared sintilimab combination therapy (pemetrexed and platinum-based chemotherapy) with the placebo combination arm in nonsquamous NSCLC. When comparing ORIENT-11 with other immunotherapy trials in first-line nonsquamous NSCLC (3–6), aside from race and Eastern Cooperative Oncology Group (ECOG), the patient populations were generally consistent (**Table 1**). A reweighted analysis of ORIENT-11 also showed consistent results, discounting race.

**Table 1:** Baseline characteristics across sintilimab, pembrolizumab, atezolizumab, and nivolumab registration studies in the first-line treatment of nonsquamous NSCLC without EGFR or ALK genomic tumor aberrations.

	ORIENT-11	KEYNOTE-189	IMpower130	IMpower150	CheckMate 9LA
PD-L1 status^a^, %					
TPS <1%	32.5	31	NA	NA	37
TPS ≥1%	67.5	63	NA	NA	57
Race, %					
Asian	100	3	2.2	9.4	NA
Non-Asian	0	97	97.8	90.6	NA
ECOG PS, %					
0	27.7	43.2	41.3	40.8	31
1	72.3	56.2	58.6	58.4	68
Median age (range)	61 (30, 75)	64 (34, 84)	64 (18, 86)	63 (31, 89)	65 (26, 86)
Male, %	76	59	59	61	70
Stage IV, %	90.9	100	100	100	100
Brain metastases, %	14.6	18	excluded	Untreated; excluded	Untreated; excluded; stable: 17
Smoking (former/current), %	65.0	88.1	90.4	84.4	86.0

ALK, anaplastic lymphoma kinase; ECOG PS, Eastern Cooperative Oncology Group Performance status; EGFR, epidermal growth factor receptor; NA, not available; NSCLC, non-small cell lung cancer; PD-1, programmed death ligand 1; TPS, tumor proportion score.

a PD-L1 expression was assessed by various assays across the studies.

The efficacy data for ORIENT-11 was consistent with other first-line immunotherapy studies in NSCLC (**Table 2**) (3–6). In the primary analysis (data cutoff: November 15, 2019) using the intent-to-treat population per blinded independent central review with a median follow-up of 8.9 months, the median duration of treatment was 7.1 and 5.5 months with the sintilimab and placebo combination arms, respectively (9). PFS was significantly improved with sintilimab when compared to the placebo arm [HR = 0.48 (95% confidence interval, CI: 0.36–0.64); *p* < 0.00001. The sintilimab combination arm provided a median PFS of 8.9 months compared with 5.0 months in the control arm [HR 0.48 (95% CI: 0.36–0.64)], which was comparable with the control arms of these other trials (**Table 2**). Similarly, the extent of improvement in PFS in the experimental arm with the addition of sintilimab was in the range of relative magnitude and hazard reduction of PFS for other approved agents for first-line nonsquamous NSCLC (**Table 2**). The sensitivity analyses supported the statistical significance of the PFS treatment effect, with observed HRs ranging between 0.48 and 0.62, each with a *p*-value of ≤0.0005. The subgroup analyses demonstrated a consistent PFS benefit across subgroups, including PD-L1 expression level and disease stage (III and IV) (9). The median OS was not yet reached in the sintilimab combination arm for the primary analysis but revealed a trend of improvement over the placebo control arm (HR 0.61, 95% CI: 0.40–0.93) (9). The updated OS analysis (data cutoff: January 15, 2021) of ORIENT-11 with a median follow-up of 22.9 months continued to demonstrate an improved OS with the addition of sintilimab to chemotherapy. Median OS was still not reached in the sintilimab arm compared with 16.8 months in the control arm (**Table 2**) (10). Despite a crossover rate in ORIENT-11 of 45.8% from the control arm to the sintilimab arm following disease progression, in line with KEYNOTE-189 (41.3%) (4), OS was still more favorable in the sintilimab combination arm [HR = 0.60 (95% CI: 0.45–0.79); *p* = 0.0003; data cutoff: January 2021].

**Table 2:** Efficacy outcomes and safety of ORIENT-11, KEYNOTE-189, IMpower130, IMpower150, and CheckMate 9LA.

Study	ORIENT-11	KEYNOTE-189	IMpower130	IMpower150	CheckMate 9LA
Combination regimen	Sintilimab + Pem + platinum (*N* = 266) *vs*. Pbo + Pem + platinum (*N* = 131)	Pembro + Pem + platinum (*N* = 410) *vs*. Pbo + Pem + platinum (*N* = 206)	Atezo + Carbo + nab-Pacl (*N* = 451) *vs*. carboplatin + nab-Pacl (*N* = 228)	Atezo + Bev + Carbo + Pacl (*N* = 356) *vs*. Bev + Carbo + Pac (*N* = 336)	Nivo + Ipi + 2 cycles platinum (*N* = 361) *vs*. 4 cycles Chemo (*N* = 358)
Efficacy					
Median PFS, in months [HR (95% CI)]	**8.9** *vs*. 5.0 [HR 0.48 (0.36, 0.64)]	**8.8** *vs*. 4.9 [HR 0.52 (0.43, 0.64)]	**7.0** *vs*. 5.5 [HR 0.64 (0.54, 0.77)]	**8.3** *vs*. 6.8 [HR 0.62 (0.52, 0.74)]	All histologies: **6.7** *vs*. 5.0[HR 0.68 (0.57, 0.82)]
Median PFS, in months [HR (95% CI)]; PD-L1 <1%	**7.3** *vs*. 5.1 [HR 0.62 (0.37, 1.03)]	**6.1** *vs*. **5.1** [HR 0.75 (0.53, 1.05)]	**6.2** *vs*. 4.7 [HR 0.72 (0.56, 0.91)]	**7.1** *vs*. 6.9 [HR 0.77 (0.61, 0.99)]	All histologies: **5.8** *vs*. 4.6 [HR 0.71 (0.53, 0.94)]
Median PFS, in months [HR (95% CI)]; PD-L1 1–49%	**7.1** *vs*. 4.8 [HR 0.51 (0.28, 0.93)]	NA [HR 0.55 (0.37, 0.81)]	**8.3** *vs*. 6.0 [HR 0.61 (0.43, 0.85)]	**8.3** *vs*. 6.6 [HR 0.56 (0.41, 0.77)]	All histologies: **6.9** *vs*. 5.3 [HR 0.69 (0.51, 0.94)]
Median PFS, in months [HR (95% CI)]; PD-L1 ≥50%	NR *vs*. 5.0 [HR 0.31 (0.19, 0.48)]	NA [HR 0.36 (0.25, 0.52)]	**6.4** *vs*. 4.6 [HR 0.51 (0.34, 0.77)]	**12.6** *vs*. 6.8 [HR 0.39 (0.25, 0.60)]	All histologies: **7.5** *vs*. 4.4 [HR 0.61 (0.42, 0.89)]
Median OS, in months [HR (95% CI)]	NR *vs*. NR[HR 0.61 (0.40, 0.93)]Updated analysis:NR *vs*. 16.8 [HR = 0.60 (0.45, 0.79)]	NR *vs*. 11.3[HR 0.49 (0.38, 0.64)]Updated analysis: **22.0** *vs*. 10.7 [HR = 0.56 (0.45, 0.70)]	**18.6** *vs*. 13.9 [HR 0.79 (0.64, 0.98)]	**19.2** *vs*. 14.7 [HR 0.78 (0.64, 0.96)]	All histologies: **14.1** *vs*. 10.7[HR 0.69 (0.55, 0.87)]Updated analysis**15.6** *vs*. 10.9[HR 0.66 (0.55, 0.80)]Nonsquamous: **17.0** *vs*. 11.9[HR 0.69 (0.55, 0.87)]
Median OS, in months [HR (95% CI)]; PD-L1 <1%	NA	NA [HR 0.59 (0.38, 0.92)]	**15.2** *vs*. 12.0 [HR 0.81 (0.61, 1.08)]	NA	All histologies: **16.8** *vs*. 9.8 [HR 0.62 (0.45, 0.85)]
Median OS, in months [HR (95% CI)]; PD-L1 1–49%	NA	NA [HR 0.55 (0.34, 0.90)]	**23.7** *vs*. 15.9 [HR 0.70 (0.45, 1.08)]	NA	All histologies: **15.4** *vs*. 10.4 [HR 0.61 (0.44, 0.84)]
Median OS, in months [HR (95% CI)]; PD-L1 ≥50%	NA	NA [HR 0.42 (0.26, 0.68)]	**17.3** *vs*. 16.9 [HR 0.84 (0.51, 1.39)]	NA	All histologies: **18.0** *vs*. 12.6 [HR 0.66 (0.44, 0.99)]
ORR (%)	**51.9** *vs*. 29.8	**47.6** *vs*. 18.9	**49.2** *vs*. 31.9	**63.5** *vs*. 48.0	All histologies: **38** *vs*. 25
DoR, in months	**NR** *vs*. 5.5	**11.2** *vs*. 7.8	**8.4** *vs*. 6.1	**9.0** *vs*. 5.7	All histologies: **11.3** *vs*. 5.6
Safety, %					
AEs related to any treatment	**99.2** *vs*. 99.2	**91.9** *vs*. 90.6	**96.2** *vs*. 92.7	**94.4** *vs*. 95.4	**92** *vs*. 88
SAEs	**28.2** *vs*. 29.8	**49.9** *vs*. 47.0	**50.7** *vs*. 37.9	**42.0** *vs*. 34.0	NR
Treatment-related SAEs	**19.2** *vs*. 16.0	**26.2** *vs*. 20.8	**23.7** *vs*. 12.9	**25.4** *vs*. 19.3	**30** *vs*. 18
AEs leading to the discontinuation of any treatment	**6.0** *vs*. 8.4	**27.7** *vs*. 14.9	**26.4** *vs*. 22	**32.6** *vs*. 24.9	NR
Treatment-related death	**0.8** *vs*. 3.1	**2.2** *vs*. 1.0	**2** *vs*. <1	**2.8** *vs*. 2.3	**2** *vs*. 2
Immune-mediated AEs	**43.2** *vs*. 36.6	**22.7** *vs*. 11.9	**45** *vs*. NR	NR	NR

Atezo, atezolizumab; Bev, bevacizumab; Carbo, carboplatin; Chemo, chemotherapy; CI, confidence interval; HR, hazard ratio; DoR, duration of response; Ipi, ipilimumab; N, number of participants; Nivo, nivolumab; NR, not reported; ORR, objective response rate; OS, overall survival; Pem, pemetrexed; Pembro, pembrolizumab; Pacl, paclitaxel; PFS, progression-free survival; Pbo, placebo; SAEs, serious adverse events; AEs, adverse events. Bold numbers indicate data from the experimental arm.

The confirmed ORR by the Independent Radiographic Review Committee was also higher with the sintilimab combination group than with the placebo (51.9 *vs*. 29.8%, respectively), and an improvement in ORR was observed with the addition of sintilimab regardless of PD-L1 TPS status. The median duration of response was not reached with sintilimab combination and was 5.5 months with placebo combination [HR = 0.54 (95% CI: 0.293–0.995)] (9).

Sintilimab demonstrated linear pharmacokinetics (PK) over the 1- to 10-mg/kg dose range, a half-life (*t*_1/2_) of approximately 14 days, and a comparable PK profile in Chinese and US patients across the body weight range of 37 to 124 kg. The population pharmacokinetics (popPK) analyses were conducted based on the data from 514 patients from four studies: the first-in-human, open-label, nonrandomized, phase 1 dose escalation and expansion study of sintilimab with or without combination therapy in advanced solid tumors (*n* = 199; CIBI308A101); the open-label, single-arm, phase 2 study of sintilimab as a single agent in classic Hodgkin lymphoma [*n* = 13; CIBI308B201 (ORIENT-1)]; the double-blind, randomized, phase 3 study of sintilimab or placebo in combination with pemetrexed and platinum-based chemotherapy in nonsquamous NSCLC [*n* = 263; CIBI308C302 (ORIENT-11 (9))]; and the multicenter, open-label, phase 1 study of sintilimab as a single agent in a Western population with advanced/metastatic solid tumors (*n* = 39; CIBI308A102).

A wide range of covariates were assessed, including body weight, race, tumor type, age, sex, creatinine clearance (37.1 to 272.2 ml/min), albumin, mild hepatic impairment (by the definition of the National Cancer Institute), ECOG performance status, and antidrug antibodies, and were found to have no clinically important effects on the PK of sintilimab. Although there was a median weight difference of 15 kg between the US and China cohorts, it only resulted in approximately 10% lower median exposure in the US population. The popPK simulations demonstrated a substantial overlap in exposure levels and was consistent with previously observed PK profiles from other immunotherapy studies in NSCLC across Asian and US populations.

A range of sintilimab doses, 1–10 mg/kg, provided near-saturation levels of PD-1 receptor occupancy (20). Therefore, the 200-mg Q3W dose regimen is likely within the plateau region of the exposure–response curve. From the exposure and efficacy data generated following the administration of the 200-mg Q3W dose regimen, there was no clinically meaningful exposure–response relationship. This flat dosing regimen of 200-mg Q3W provided a statistically significant and clinically meaningful improvement in PFS compared to the placebo arm and a tolerable safety profile. The totality of the PKPD, efficacy, and safety data supported the 200-mg Q3W as the appropriate dosing regimen across both Asian and US populations.

Noncatabolic pathways, such as hepatic metabolism, generally aid in the clearance of small molecules. Subsequently, interracial differences of the small molecules’ PK can result due to polymorphic differences in the hepatic cytochrome P450 isoenzymes between Asians and non-Asians (28). In contrast, sintilimab, as a monoclonal antibody, is administered by IV infusion and eliminated by catabolic pathways, subsequently having minimal ethnic effects such as metabolism and absorption (20, 28). Body weight is a major ethnic difference between Asian and Western populations and warrants consideration in the context of dosing (29). However, previous analyses comparing 12 monoclonal antibodies have demonstrated that fixed dosing and body size-based dosing with monoclonal antibodies resulted in systemic exposures which were comparable (29). This further supports the rationale of using the 200-mg 3-weekly dosing regimen for sintilimab across both Asian and non-Asian populations. To summarize the popPK findings described above, sintilimab was not sensitive to race, in line with other anti-PD-1/L1 therapies which have demonstrated comparable pharmacokinetics across Asian and Western populations (1, 13, 30–34). This further reiterates the lack of ethnic differences between Asian and Western populations in regards to the PK of sintilimab.

In phase 3 studies, the safety profiles for checkpoint inhibitors have been generally consistent across studies and also between Asian and Western populations (4, 12, 13, 25, 26, 31, 32, 34–36). The safety profile of sintilimab in the combination with pemetrexed and platinum (9) is also largely consistent with that observed across other global studies of PD-1/L1 inhibitors, with similar patterns and incidences of adverse events (AEs) (**Table 2**). The subtypes and rates of immune-related adverse events (irAEs) in the sintilimab combination arm were overall similar to irAEs observed across the class of anti-PD-1/PD-L1 therapies, including immune-mediated pneumonitis (3, 4, 6). In addition, the results of a meta-analysis which included 10 first-line immunotherapy combination studies in NSCLC indicated that sintilimab plus chemotherapy had comparable safety and irAEs with other immunotherapy combinations (37). This meta-analysis by Liu et al. revealed a relatively narrow range for the incidence of immune-mediated AEs, with sintilimab chemotherapy treatment that was on the lower end of the spectrum, demonstrating comparable irAE profiles across the different checkpoint inhibitor combination studies (37). Furthermore, the majority of irAEs observed with sintilimab therapy have been lower grade, with less than 6% incidence of grade ≥3 irAEs (9).

The high and consistent median relative dose intensity [sintilimab (97.1%); placebo (97.4%)] in ORIENT-11 demonstrated sintilimab’s tolerability in nonsquamous NSCLC. The incidence of treatment-emergent adverse events leading to discontinuation [5.3% (sintilimab) *vs*. 6.9% (placebo)] or death [2.3% (sintilimab) *vs*. 6.9% (placebo)] was low and fairly comparable between the treatment arms.

Although only a limited number of Western participants (*n* = 39) were included in the sintilimab overall safety analysis, it was supplemented by data from 1,045 patients including a variety of advanced solid tumor types as well as different lines of prior therapy. This evaluation of pooled analyses suggested that the safety profile of sintilimab may be comparable between Asian and US participants with no notable safety findings or new safety concerns. Chemotherapy-related toxicities were consistent between the treatment arms, and the addition of sintilimab did not appear to cause an interruption or discontinuation of chemotherapy administration. Furthermore, the immune-mediated AEs observed across the treatment arms were mostly manageable with study drug dose modification, supportive care, and corticosteroid use.

### Extrinsic Factors

The extrinsic factors of PD-L1 testing and stage at diagnosis vary across the world, the latter of which reflects differences in access; however, the ORIENT-11 study in China used the same staging system [based on American Joint Committee on Cancer (AJCC) as described in the 8th edition of the TNM classification by the International Association for the Study of Lung Cancer) (9, 38) and PD-L1 testing (PDL1 IHC 22C3 pharmDx, Agilent Technologies) (9) as used in the United States. Both countries used similar pathology guidelines, diagnosis, inclusion/exclusion criteria, treatment (including supportive and prior medications), follow-up (*e*.*g*., scans and intervals), staging system, and PD-L1 biomarker testing. In addition to including patients with stage IV NSCLC, the study also permitted patients with stage III unresectable NSCLC and not suitable for chemoradiation with a curative intent, thereby generating additional data in patients with advanced disease. At the time of the study design, access to the second-line immunotherapy treatment was limited in China. However, in the control arm of the study, patients could be sequenced to second-line sintilimab monotherapy, contingent upon disease progression, making second-line immunotherapy accessibility comparable to those of studies run in the United States and Europe. Furthermore, there was consistency in terms of medical practice and the standard-of-care first-line treatment options of immune checkpoint inhibitors with platinum-based chemotherapy backbone (39–41).

### Quality Standards of ORIENT-11

ORIENT-11 was a well-designed study conducted in China in accordance with the ethical guidelines established from the Declaration of Helsinki and the ICH E (6) Good Clinical Practice (GCP) and therefore performed to Western standards. The ORIENT-11 investigators were competent and well-qualified to treat NSCLC and were predominantly GCP-certified medical oncologists. The inclusion and exclusion criteria were well-defined and in line with KEYNOTE-189 and CheckMate 9LA, with a similar distribution of patients across the PD-L1 expression strata (**Table 1**). The clinical trial employed screening tactics which effectively excluded patients who had driver mutations. Furthermore, the trial design for ORIENT-11, when initiated in 2018, was consistent with the treatment guidance in China for first-line nonsquamous NSCLC without epidermal growth factor receptor (*EGFR*) mutations or *ALK* fusions (39–41). In addition to this requirement, the use of RECIST for response evaluation, frequency of scans, central read/investigator read, use of CTCAE, and AE management (including supportive medications) were similar between KEYNOTE-189 and ORIENT-11, further demonstrating that the ORIENT-11 study met the standards for study quality, data quality, and ethics suitable for applicability to Western geographies (9).

## Discussion

When considering cultural and environmental extrinsic factors, the key clinical practice components such as PD-L1 testing methods and staging system use (AJCC 8^th^ edition) were similar between the US and China. Regarding genetic and physiologic intrinsic factors, the ORIENT-11 patient population was generally consistent with those of other immunotherapy studies in first-line nonsquamous NSCLC performed in the US, with the exception of race and ethnicity. Although *EGFR* mutations have been previously found to occur with increased frequency in Asian populations (~35%) when compared to Western populations (~10%) (23, 42), patients with these mutations were excluded from the ORIENT-11 trial (9). In terms of effectiveness and safety of the medicine, the outcomes of ORIENT-11 with sintilimab were consistent with that of other PD-1/L1 inhibitors developed in first-line nonsquamous NSCLC in largely Western populations (4, 9).

The results for individual immune checkpoint inhibitor studies have shown similar efficacy, popPK, and safety outcomes in Asian *vs*. non-Asian patients, which is also supported by the recent results of the FDA meta-analyses (27). Sintilimab is in the same class of immunotherapy agents as pembrolizumab, nivolumab, and atezolizumab. In evaluating the key extrinsic and intrinsic factors outlined by the ICH (including treatment guidelines, patient demographics, and popPK), the information suggests that the clinical data from ORIENT-11 can be extrapolated to patients outside of China. The study population of ORIENT-11 Chinese patients provides data which can reasonably be expected to be replicated in a typical US population within the target population of the trial. The PK and safety outcomes for sintilimab are also consistent with other NSCLC immunotherapy studies and comparable across Asian and Western patient populations. The outcomes of the control arm of ORIENT-11 corresponded with those observed in Western patients, and the high crossover to the sintilimab arm revealed equivalence of clinical practice. Taking all this into account, the intrinsic (age, race, and tumor type) and extrinsic factors did not appear to have a clinically important impact on the PK or safety in ORIENT-11.

In conclusion, PD-1/PD-L1 inhibitors have demonstrated similar outcomes in Asian and Western patients. The ORIENT-11 study provides compelling data in the Chinese patients that are applicable to the US population; however, we do acknowledge that there may be additional unknown and unmeasured factors that could potentially affect the questions posed herein. Redundant repetition of studies should be avoided, and it would also be unethical to do so given the current standard of care. In evaluating the totality of extrinsic and intrinsic factors, this body of evidence supports the use of sintilimab combined with platinum and pemetrexed as first-line therapy for the treatment of nonsquamous NSCLC in US populations.

## Data Availability Statement

Eli Lilly and Company provides access to all individual data collected during the trial, after anonymization, with the exception of pharmacokinetic, genomic, or genetic data. Data are available to request 6 months after the indication studied has been approved in the US and EU and after primary publication acceptance, whichever is later. No expiration date of data requests is currently set once data are made available. Access is provided after a proposal has been approved by an IRC identified for this purpose and after receipt of a signed data sharing agreement. Data and documents, including the study protocol, statistical analysis plan, clinical study report, and blank or annotated case report forms, will be provided in a secure data sharing environment. For details on submitting a request, see the instructions provided at www.vivli.org.

## Ethics Statement

ORIENT-11 was a well-designed study conducted in China in accordance with the ethical guidelines established from the Declaration of Helsinki and the ICH E (6) Good Clinical Practice (GCP) and therefore performed to Western standards. The ORIENT-11 investigators were competent and well-qualified to treat NSCLC and were predominantly GCP-certified medical oncologists. The patients/participants provided their written informed consent to participate in this study.

## Author Contributions

Interpretation of the data for the work: SL, YL, VS, LZ, MN, KG, MS, and DF. Drafting of the work: SL, KG, and DF. Critical revision of the work: SL, YL, VS, LZ, MN, KG, MS, and DF. Conception: SL, LZ, MS, and DF. Design: SL and LZ. Acquisition: LZ. Analysis of data: SL, LZ, MN, MS, and DF. All authors contributed to the article and approved the submitted version.

## Funding

This work was supported by Eli Lilly and Company.

## Conflict of Interest

SL: advisory board/consultant for Amgen, AstraZeneca, Bayer, Beigene, Blueprint, Bristol-Myers Squibb, Eisai, Elevation Oncology, Genentech/Roche, Gilead, Guardant Health, Janssen, Jazz Pharmaceuticals, Lilly, Merck/MSD, Novartis, Regeneron, Sanofi, Takeda, and Turning Point Therapeutics. Research funding (to institution): Alkermes, Bayer, Blueprint, Bristol-Myers Squibb, Elevation Oncology, Genentech, Lilly, Merck, Merus, Pfizer, Rain Therapeutics, RAPT, Turning Point Therapeutics. MS: fees for non-CE services received directly from an ineligible entity or their agents (*e*.*g*., speakers’ bureaus) from Genetech, Astra Zeneca, Eli Lilly and Company, Blueprint, Janssen, GI Therapeutics, Amgen, Merck, and Takeda; contracted research from Astra Zeneca, Spectrum, BeiGene, Cullinan, Morati, Daiichi, Sankyo, and Pfizer. MN: advisory boards for AstraZeneca, Daiichi Sankyo, Takeda, Novartis, EMD Serono, Janssen, Pfizer, Eli Lilly and Company, and Genentech; consultant for Caris Life Sciences (virtual tumor board); speaker for Blueprint Medicines and Takeda; and reports travel support from An Heart Therapeutics. LZ: grants from AstraZeneca, Bristol-Myers Squibb, and Pfizer. YL, VS, and DF are employees of Eli Lilly and Company with stock options. KG is an employee of Eli Lilly and Company.

The remaining authors declare that the research was conducted in the absence of any commercial or financial relationships that could be construed as a potential conflict of interest.

## Publisher’s Note

All claims expressed in this article are solely those of the authors and do not necessarily represent those of their affiliated organizations, or those of the publisher, the editors and the reviewers. Any product that may be evaluated in this article, or claim that may be made by its manufacturer, is not guaranteed or endorsed by the publisher.
